# Efficacy of supervised self-reduction vs. physician-assisted techniques for anterior shoulder dislocations: a systematic review and meta-analysis

**DOI:** 10.1186/s12891-024-07379-0

**Published:** 2024-05-11

**Authors:** Amir Human Hoveidaei, Mahdi Dankoub, Mohammad Mehdi Mousavi Nasab, Amin Nakhostin-Ansari, Alireza Pouramini, Shayan Eghdami, Fatemeh Mashaknejadian Behbahani, Moein Zangiabadian, Bijan Forogh

**Affiliations:** 1 Neuromusculoskeletal Research Center, Department of Physical Medicine and Rehabilitation, School of Medicine, Iran University of Medical Sciences, Tehran, Iran; 2 International Center for Limb Lengthening, Rubin Institute for Advanced Orthopedics, Sinai Hospital of Baltimore, Baltimore, MD USA; 3 School of Medicine, Tehran University of Medical Sciences, Tehran, Iran; 4 School of Medicine, Shahid Beheshti University of Medical Sciences, Tehran, Iran; 5 Sports Medicine Research Center, Neuroscience Institute, Tehran University of Medical Sciences, Tehran, Iran; 6 Research Committee, Iran university of Medical Sciences, Tehran, Iran; 7 Endocrinology and Metabolism Research Center, Institute of Basic and Clinical Physiology Sciences, Kerman University of Medical Sciences, Kerman, Iran; 8 Neuromusculoskeletal Research Center, Department of Physical Medicine and Rehabilitation, School of Medicine, Firoozgar General Hospital, Iran University of Medical Sciences, Tehran, Iran

**Keywords:** Shoulder dislocation, Reduction, Pain, Success rate, Efficacy

## Abstract

**Background and objective:**

Reduction manipulation using self-reduction procedures such as Stimson, Milch, and Boss-Holtzach should be easy and effective and also require less force, pain medication, and outside assistance. This technique should not cause damage to arteries, nerves, or shoulder joint components. Self-reduction is straightforward and can be done in clinics, making it ideal for people who suffer from shoulder joint dislocation frequently. The goal of this study is to compare the effectiveness of supervised self-reduction procedures vs. physician-assisted treatments in the treatment of anterior shoulder dislocations.

**Method:**

We conducted a comprehensive search on PubMed, Scopus, Web of Science, and Cochrane up to March 22, 2023, using the Preferred Reporting Items for Systematic Reviews and Meta-Analyses (PRISMA) checklist. Relevant articles were reviewed, with a focus on studies comparing supervised self-reduction techniques to physician-assisted techniques in cases of anterior shoulder dislocation.

**Results:**

Four papers in all were included in the meta-analysis. One prospective trial, one case-control study, one randomized clinical trial, and one retrospective trial made up these studies. The studies involved 283 patients in the physician-assisted group and 180 patients in the supervised self-reduction group. They were carried out in four European countries: Italy, Germany, Portugal, and Spain. The success rate of supervised self-reduction techniques was significantly higher, with an odds ratio of 2.71 (95% CI 1.25–5.58, p-value = 0.01). Based on the Visual Analog Scale (VAS) score, the physician-assisted group reported significantly higher maximum pain, with a mean difference of 1.98 (95% CI 1.24–2.72, p-value < 0.01). The self-reduction approaches exhibit shorter reduction time in comparison to physician-assisted groups. In addition, the self-reduction groups do not document any complications. Based on the GRADE system, the level of assurance in the evidence was high.

**Conclusion:**

Supervised self-reduction techniques outperform in terms of success rate and reduction-related maximum pain. These techniques could be used as an effective first-line treatment for anterior shoulder dislocation, potentially reducing the need for analgesics and emergency room visits.

**Supplementary Information:**

The online version contains supplementary material available at 10.1186/s12891-024-07379-0.

## Introduction

Shoulders account for over 50% of major joint dislocations. Most occurrences (90–95%) include anterior shoulder dislocation [1, 2]. This dislocation occurs between 11.2 and 23.9 times per 100,000 person-years in the US, according to studies [3, 4].

Dislocation leads to a complete disruption of communication between the surfaces of the joint, along with harm to the stabilizing components. The glenoid cavity and humerus work together to form the shoulder’s articular surfaces. A head-to-cavity ratio ranging from 4:1 to 3:1 offers significant mobility and stability, but it also increases the risk of re-dislocation following a shoulder dislocation [5].

There are various procedures for reducing shoulder dislocation. Some of these treatments require the assistance of a physician, such as traction-countertraction and scapular manipulation [6]. Other techniques can be performed by the patient, such as the Stimson, Milch, and Boss-Holtzach methods [7]. Due to the prevalence of shoulder dislocations in remote areas during sports or outdoor activities, where immediate medical assistance may not be accessible, it is crucial to minimize the time before attempting reduction. Therefore, there is a demand for a straightforward, efficient, and rapid manipulation technique that can serve as the primary treatment for anterior shoulder dislocations. This approach aims to reduce healthcare expenses and minimize the time required for reduction [8]. It should also require less assistance, analgesia, and force, and it should not hurt the arteries, nerves, or shoulder joint tissues like the labrum and humerus head [9].

Self-reduction is an optimal approach for patients who frequently suffer from shoulder joint dislocations due to its simplicity and suitability for many settings, including clinics [10, 11]. Nevertheless, these procedures might lead to problems such as humeral fracture or nerve injury [12]. Compromise of the axillary nerve occurs in more than 40% of dislocations but often resolves with reduction. Clinically important fractures occur in about 25% of dislocations [13]. Nevertheless, some authors speculated that the utilization of self-reduction strategies by patients, while being overseen by a physician, might potentially be regarded as a “supervised self-reduction” technique that is both efficacious and straightforward to execute [14].

To date, no systematic reviews have directly compared the efficacy of self-reduction versus physician-assisted techniques, and their worth is debatable. As a result, comparing their effectiveness is crucial in order to select the optimum technique. The purpose of this research is to assess the effectiveness of self-reduction versus physician-assisted treatment for anterior shoulder dislocations.

## Methods

The design and methods used for this review comply with CRD’s Guidance for Undertaking Reviews in Healthcare [15] and are reported in line with Preferred Reporting Items for Systematic Reviews and Meta-Analyses 2020 (PRISMA 2020) [16]. This systematic review has been registered in the International Prospective Register of Systematic Reviews (PROSPERO) under the registration ID: CRD42022302723.

### Search strategy

Based on the search strategy outlined in (Appendix S1), we performed a comprehensive search on the 15th of May 2022 in MEDLINE (PubMed), Scopus, Cochrane Central Register of Controlled Trials, and Web of Science. There were no restrictions on time frame, geography, or language. In addition, we conducted a thorough search for relevant studies by examining both forward and backward citations of the included studies. It is important to note that we also updated the search process on the 22nd of March 2023 to ensure the inclusion of any recent studies.

### Eligibility criteria

This study aims to incorporate studies on patients with anterior shoulder dislocation, regardless of age, gender, or ethnicity. The emphasis was on first-time dislocations as well as chronic patients with repeating episodes. In this study, supervised self-reduction procedures such as Boss-Holtzach-Matter, Milch, and Stimson were compared to a physician-assisted technique utilizing traction-counter traction or scapular manipulation. Our meta-analysis included only randomized clinical trials, prospective trials, case-control studies, and retrospective trials. Case reports, case series, letters, correspondence, and commentary were all excluded,

Citations found through searches are integrated into the Rayyan QCRI app [17], a web-based tool that uses natural language processing, artificial intelligence, and machine learning technologies to expedite the screening of record titles and abstracts. Duplicates were identified and eliminated by Endnote. The titles and abstracts of the chosen studies that matched the established eligibility criteria are next thoroughly examined by two distinct reviewers (MD, MMMN). Following that, the same reviewers separately evaluated the full text of all potentially eligible recovered records. When there was disagreement, a third author [16] was consulted for their opinion.

The reasons for exclusions were documented and are included in a table in the final evaluation (Appendix S2). In the results, we report the study selection process utilizing PRISMA flow diagrams of study selection.

### Data extraction

To gather information from articles, two authors (MD, MMMN) worked independently. They collected the study’s title, the name of the corresponding author, demographic details (such as participant age, gender, and sample size), the type of study, the intervention used, inclusion criteria, and outcome measures (such as success rate, pain reduction, and reduction time). They entered this information into pre-designed data extraction forms in Microsoft Excel spreadsheets (version 2016, created by Microsoft Corporation in the USA). If necessary, they addressed any issues by consulting a third review author [16].

### Risk of bias

Two authors (MD, MMMN) independently evaluated all the eligible included studies and recorded supporting information for judgments of risk of bias in six domains of random sequence generation (selection bias for controlled trials), allocation concealment (selection bias for controlled trials), blinding of participants and personnel (performance bias), blinding of outcome assessment (detection bias), incomplete outcome data (attrition bias) and selective reporting (reporting bias) according to the Cochrane “Risk of bias” tool and attributed each domain to be of “low risk”, “high risk”, or “unclear risk” of bias for each article as reported in (S1 table) [18].

To assess the presence of publication bias across different studies, we performed a contour-enhanced funnel plot and Galbraith plot. The funnel plot utilized Fisher’s z-transformed correlation to examine any potential publication bias visually. To enhance the interpretability of the plot, contour lines corresponding to commonly recognized levels of statistical significance (p-value = 0.01, 0.05, and 0.1) were included. Additionally, we conducted a test to determine if there was any asymmetry in the funnel plot, which could indicate the presence of publication bias. Whenever two authors had differing opinions regarding the quality of studies, they resolved these disagreements by engaging in discussions or seeking input from a third author [16].

### Statistical analysis

By the use of STATA version 17.0, we combined and compared the outcome data between the physician-assisted vs. self-reduction techniques for each extracted outcome. Pooled estimates of odd ratios (ORs) for dichotomous outcomes and mean differences (MDs) for maximum pain intensity were calculated with 2-sided 95% confidence intervals (CIs) using a random-effects model and restricted maximum likelihood estimator.

To assess the heterogeneity between studies, we utilized the chi-square statistic, its associated P value, and the I2 statistic. We considered a P value of less than 0.1 and an I2 value greater than 40% as indicators of substantial between-study heterogeneity. If significant statistical or clinical heterogeneity was detected, we employed a random-effects model for conducting the meta-analysis.

## Results

**Fig. 1:**
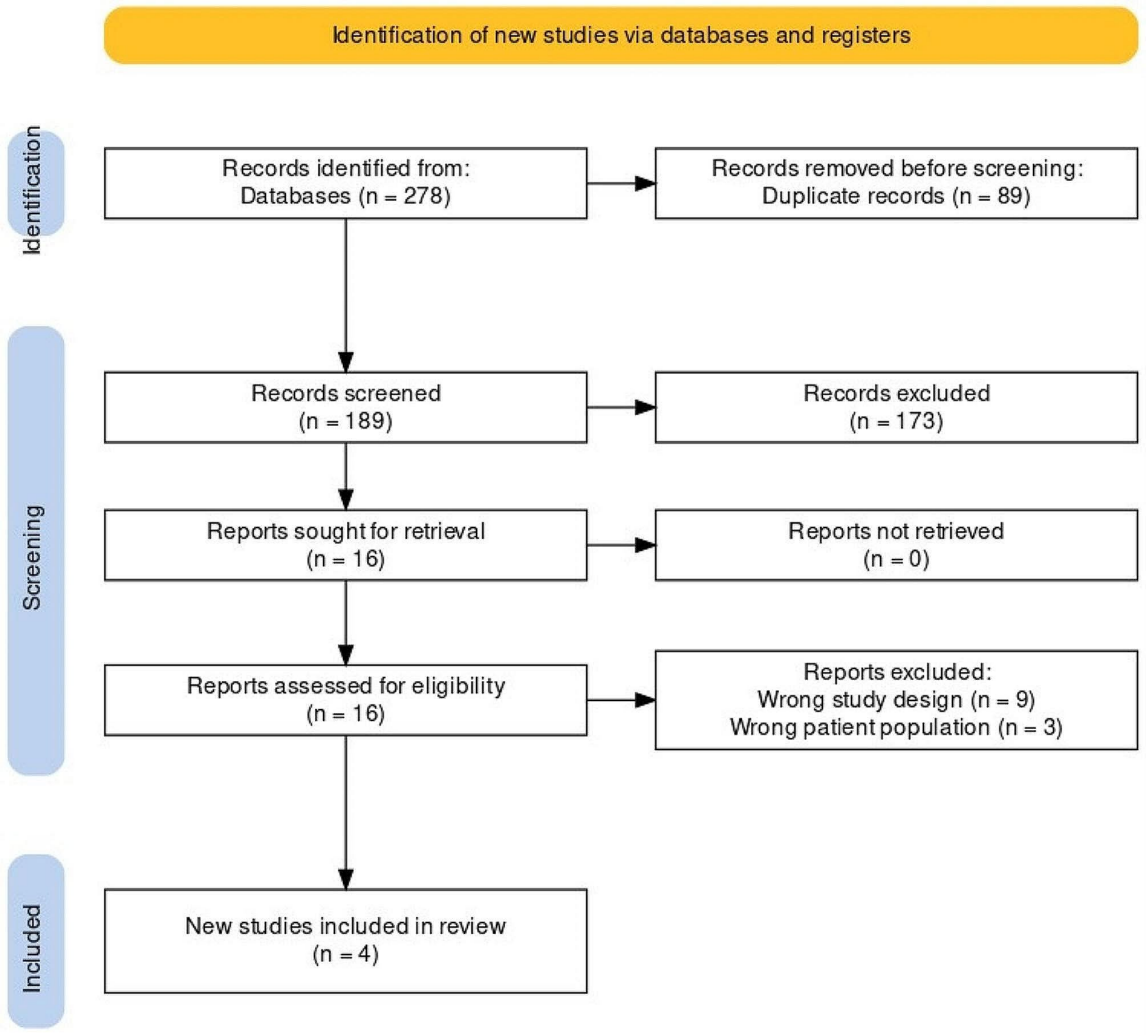
PRISMA flow diagram of study

In our search for this review, we found 278 records (27 CENTRAL, 46 Scopus, 165 MEDLINE through PubMed, and 40 Web of Science). After removing 89 duplicates, we screened the titles and abstracts of 189 records. At that point, another 173 records were eliminated, leaving only 16 for full-text evaluation. After reviewing the full text of the records, we eliminated 12 studies. Four studies that met the criteria were included in the meta-analysis [2, 8, 14, 19]. Figure 1 shows the PRISMA flow diagram, which provides a detailed summary of the search results.

All studies were published from 2014 to 2022 consisting of 283 patients in the physician-assisted group and 180 patients in the self-reduction group from four different countries of Spain [8], Portugal [19], Italy [14], and Germany [2] (Table 1).

**Table 1:** Characteristics of included studies

First Author	Published year	Study design	Country	Supervised Self-Reduction Techniques % (Success/Total)	Physician Assisted techniques
Marcano Fernandez et al. [18]	2018	Randomized clinical trial	Spain	• BHM Technique: 76.6% (23/30)	• Spaso: 66.6% (20/30)
Silva et al. [19]	2022	Prospective trial	Portugal	• Davos: 87.5% (35/40)	• Traction/countertraction: 85.0% (34/40)
Turturro et al. [12]	2014	Prospective case– control	Italy	• Kocher: 98.3% (60/61)	• Traction/countertraction: 88.0% (155/176)
Wirbel et al. [2]	2014	Retrospective trial	Germany	• BHM Technique: 71.4% (25/35) • Kocher: 64.2% (9/14)	• Traction/countertraction: 32.4% (12/37)

### Success rate

The overall pooled odds ratio of supervised self-reduction vs. physician-assisted techniques success rate in included studies was found to be 2.71 (95% CI 1.2 5- 5.58, P-value = 0.01, and with a low degree of statistical heterogeneity I^2^: 35.47%) according to a random effects model (Fig. 2).

**Fig. 2A:**
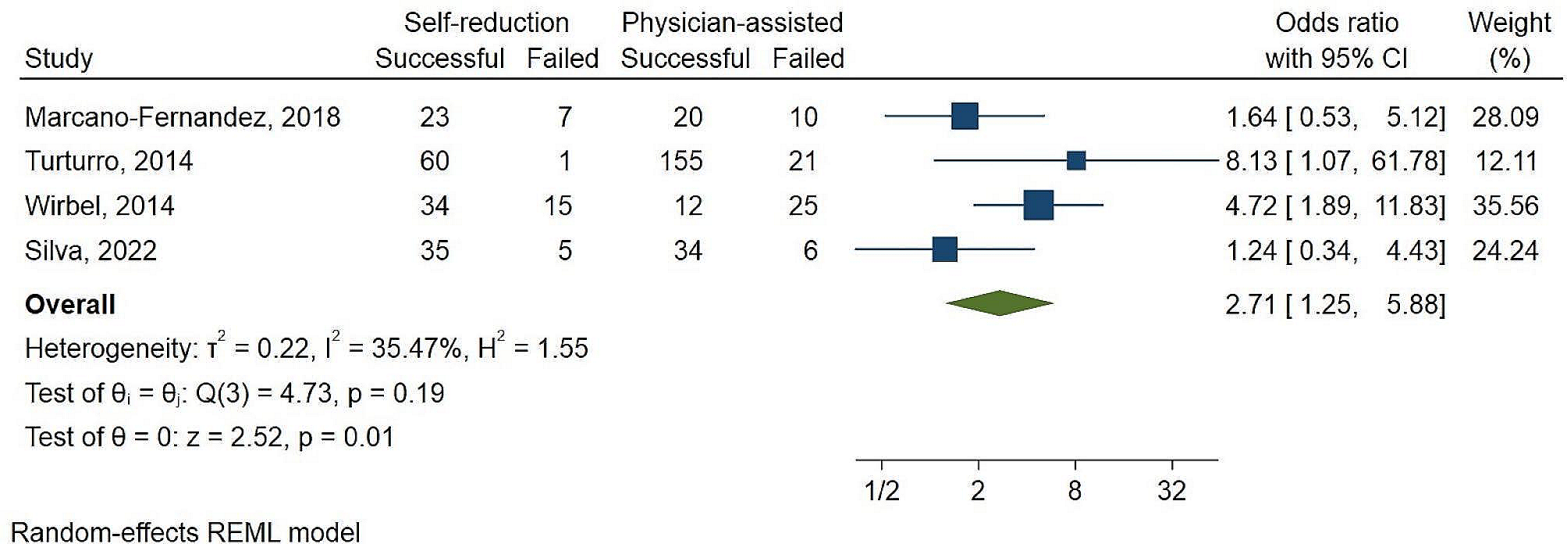
The forest plot comparing successful reduction of supervised self-reduction vs physician-assisted techniques in shoulder dislocation.

**Fig. 2B:**
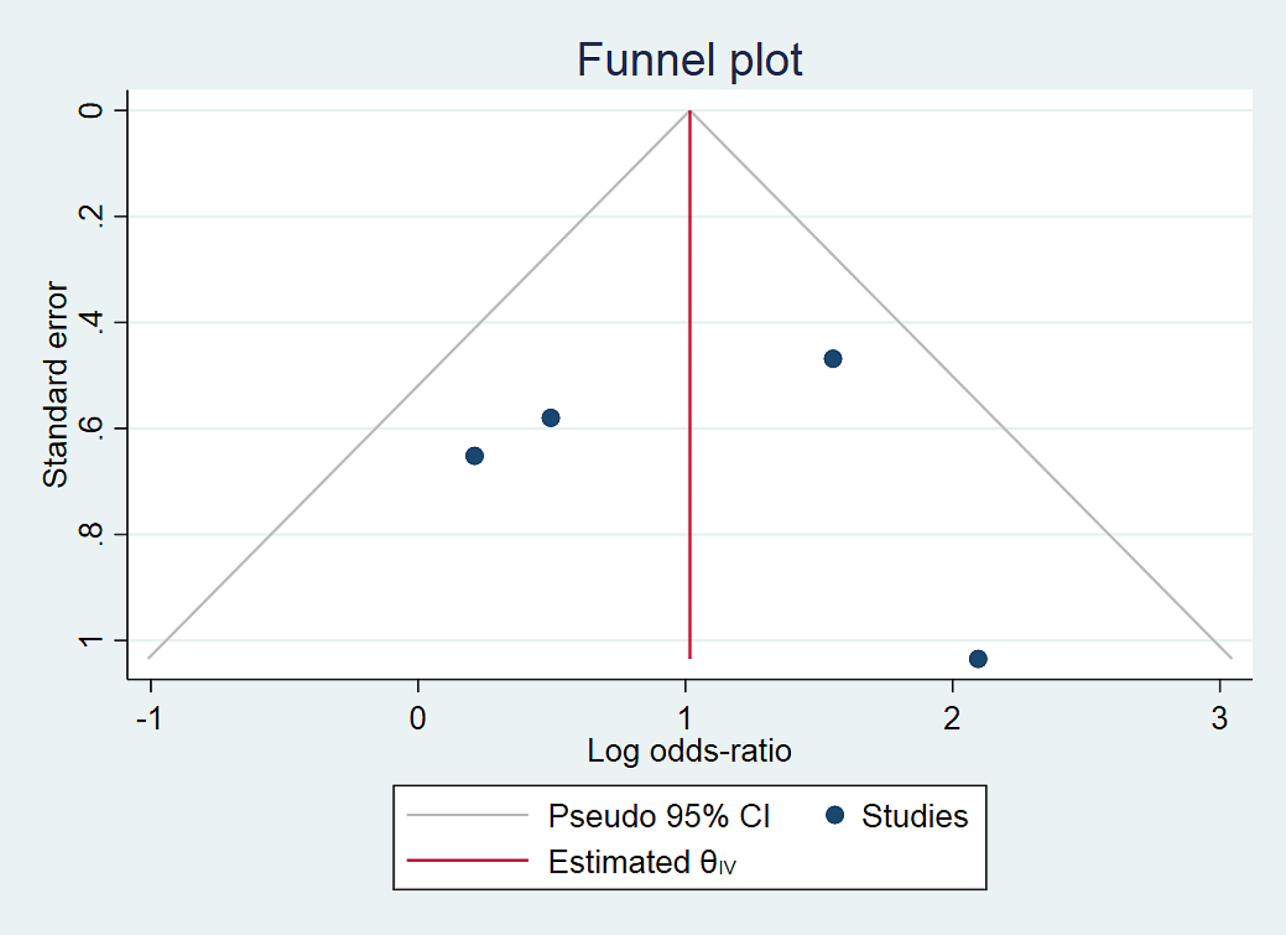
The funnel plot comparing successful reduction of supervised self-reduction vs. physician-assisted techniques in shoulder dislocation

**Fig. 2C:**
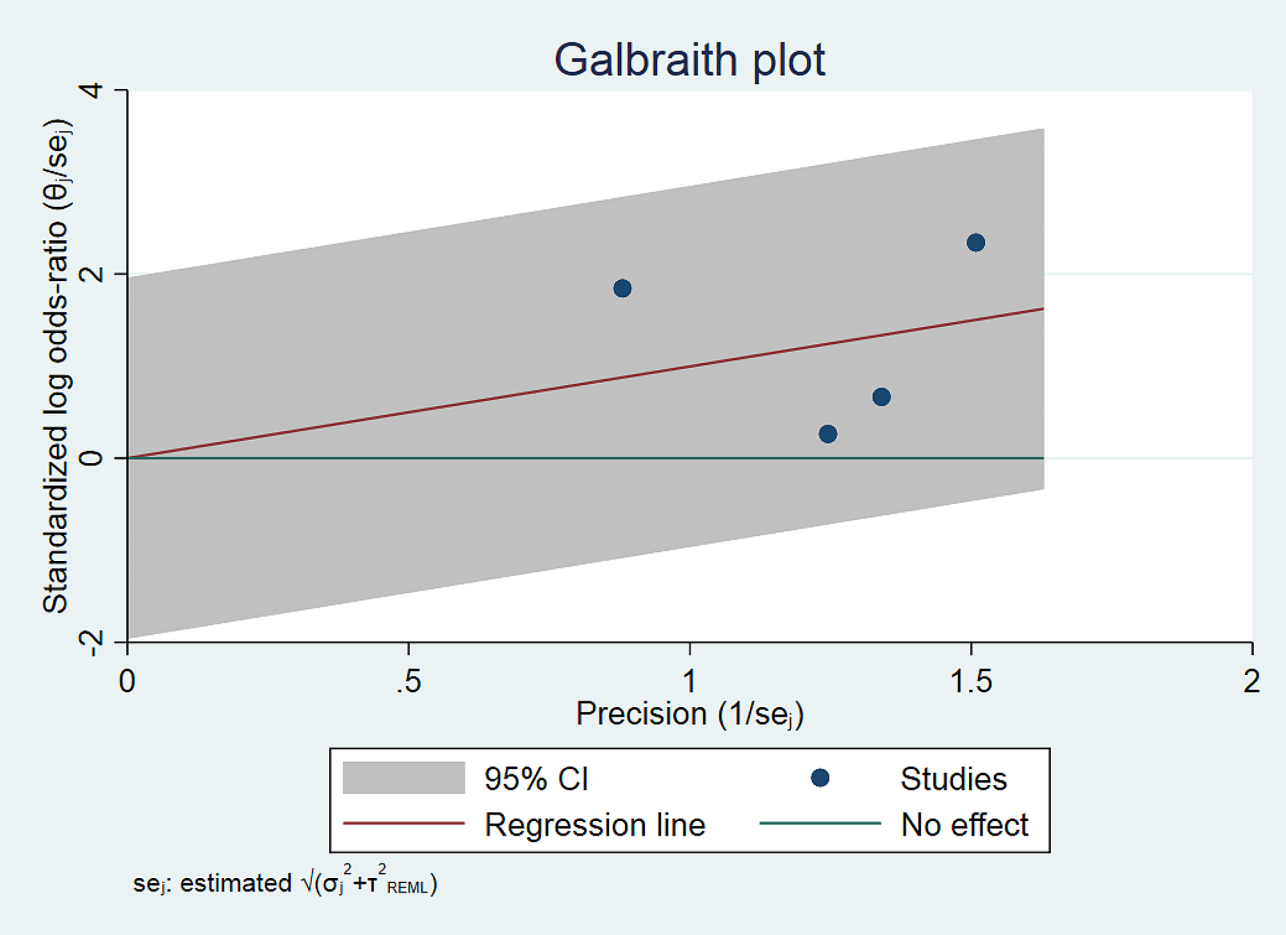
The Galbraith plot comparing the successful reduction of supervised self-reduction vs. physician-assisted techniques in shoulder dislocation

### Reduction pain

Pooled analysis of two studies with a combined sample of 140 patients indicated a significant (P-value < 0.01) mean difference of maximum pain during anterior shoulder reduction between groups of 1.98 (95% CI 1.24–2.72, I^2^: 0.0%) that indicate a low degree of statistical heterogeneity (Fig. 3).

**Fig. 3A:**
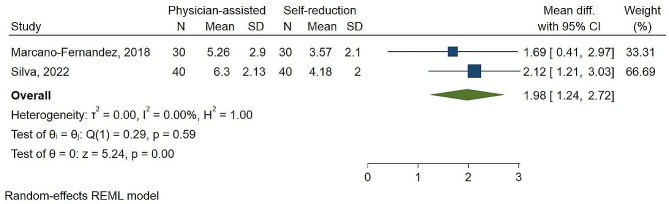
The mean difference of maximum pain experienced during anterior shoulder reduction with physician-assisted vs. self-reduction techniques according to VAS score

**Fig. 3B:**
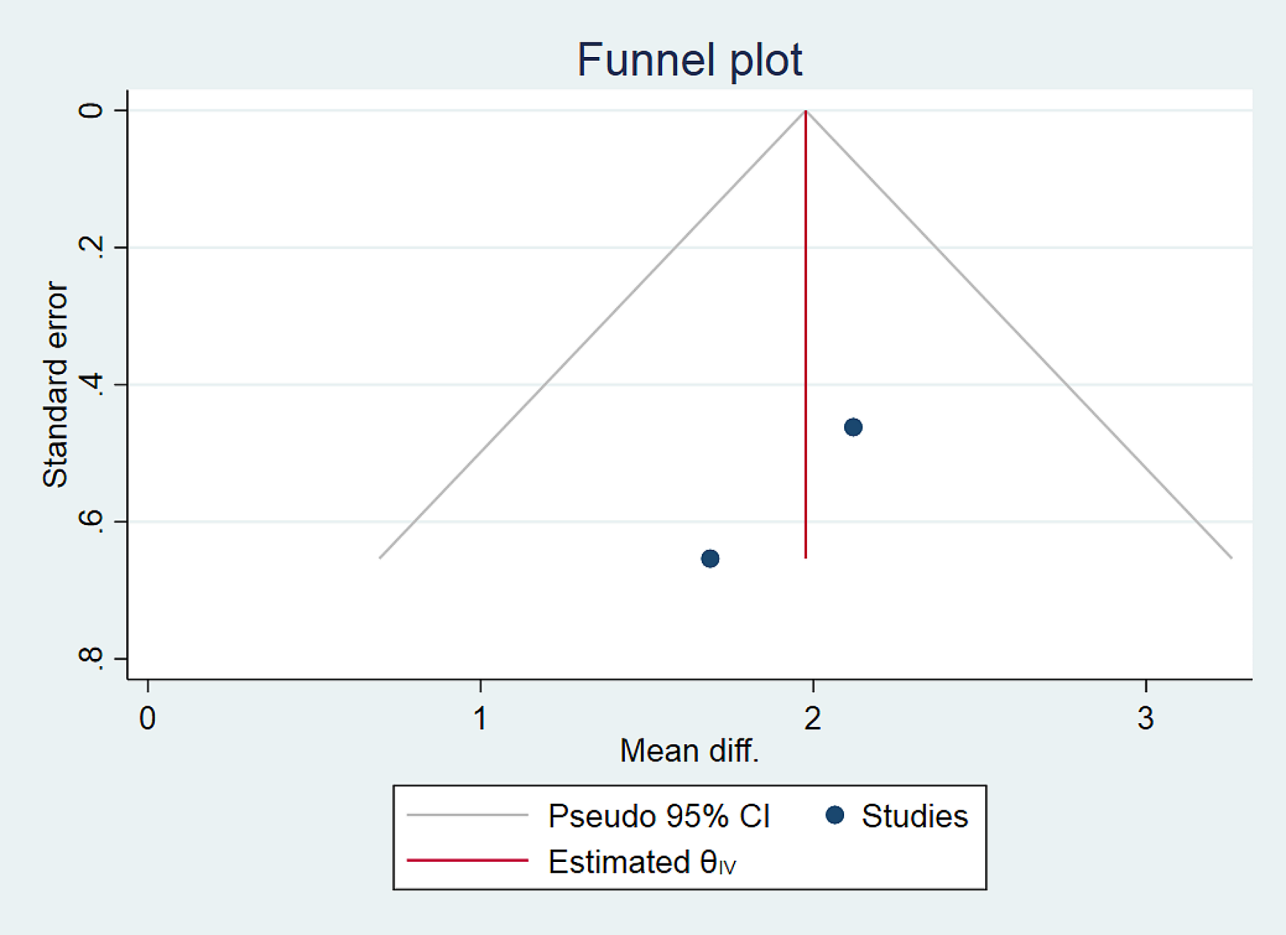
The funnel plot of mean difference of maximum pain experienced during anterior shoulder reduction with physician-assisted vs. self-reduction techniques according to VAS score

**Fig. 3C:**
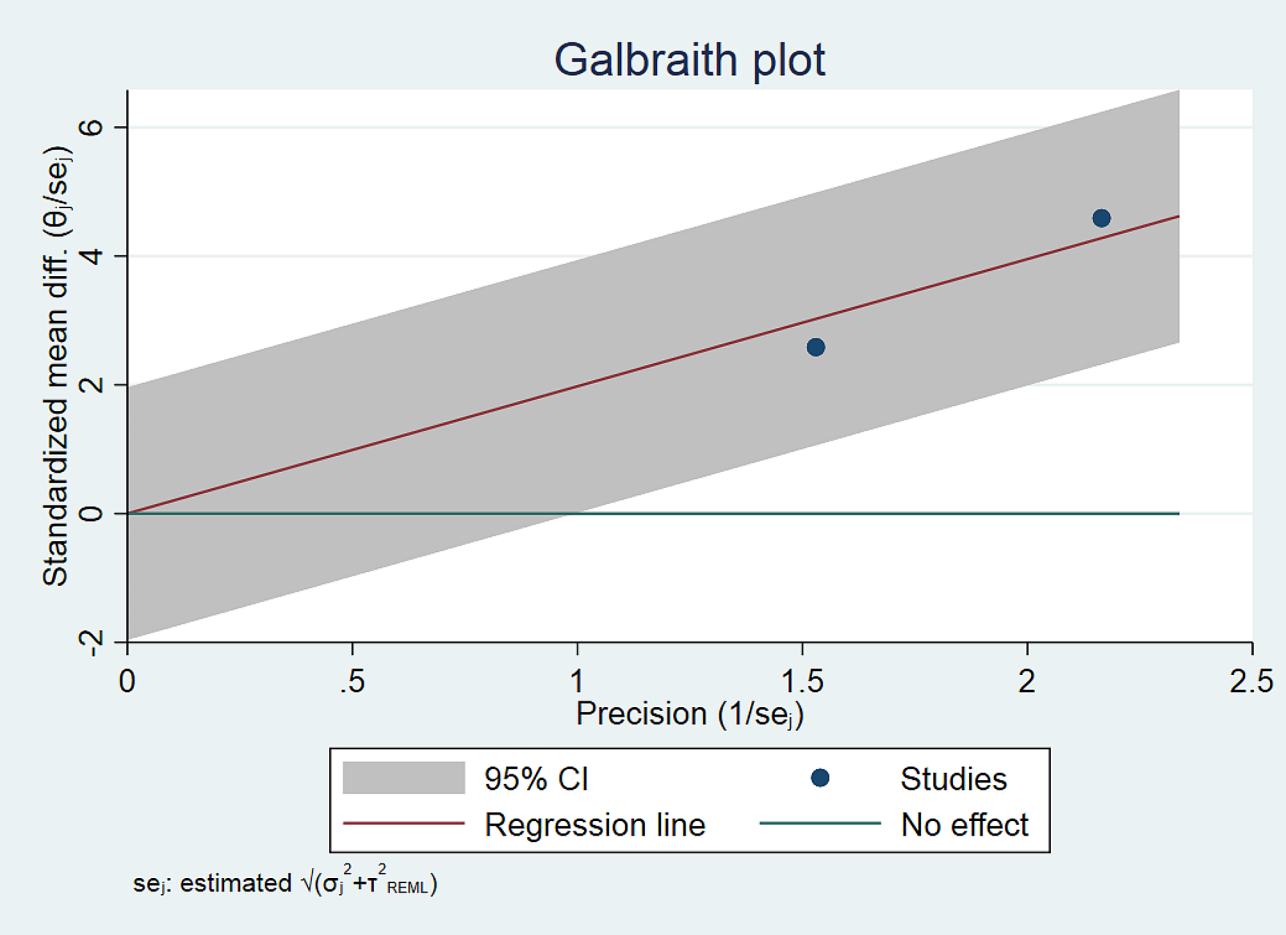
The Galbraith plot of mean difference of maximum pain experienced during anterior shoulder reduction with physician-assisted vs. self-reduction techniques according to VAS score

Marcano-Fernandez et al. [8] reported that the physician-assisted group experienced significantly higher overall pain (5.26 ± 2.9 vs. 3.57 ± 2.1; *P* = 0.047).

### Reduction time

Two studies report reduction time that in both of them, physician-assisted needs more time. In the Marcano-Fernandez study [8] reduction time was higher in the physician-assisted group (mean of 105 s with a range of 10 to 660) vs. the self-reduction group (mean of 90 s with a range of 5 to 600) with p-value = 0.608. Turturro et al. [14] also report mean procedural time of 4ʹ36ʺ (3ʹ–12ʹ) and 5ʹ45 (4ʹ02ʺ–15ʹ47ʺ) for self-reduction and physician-assisted group respectively. The two included articles presented the range and mean. Converting this data to mean and SD can introduce bias due to the limited sample size of the papers.

### Complications

Three studies report no acute complication following shoulder reduction in either intervention group [8, 14, 19].

Table 2 summarizes the outcomes of the meta-analysis according to the GRADE criteria.

**Table 2:** Summary of findings based on GRADE Approaches

Outcome	Relative effect (CI 95%)	Intervention vs. comparator mean difference (CI 95%)	No of participants (studies)	Quality of the evidence (GRADE)	Comments
Success rate	OR 2.71 (1.25–5.58)	-------	463(4)	High	-------
Reduction pain	------	Mean 1.98 (1.4–272)	140(2)	High	The low mean reduction pain indicates more efficacy of Self- reduction techniques

Patient or Population: Patients with anterior shoulder dislocation

Intervention: Self-Reduction techniques

Comparison: Physician-assisted techniques

### The quality of studies included

The assessment of the quality of the studies included in the study was provided in table S1.

### Publication Bias

The publication bias was evaluated using funnel plots and Galbraith plots for each outcome in the meta-analysis (Figs. 2B and C and 3B and C).

## Discussion

To the best of our knowledge, this is the first meta-analysis to compare the effectiveness of supervised self-reduction procedures with physician-assisted techniques for anterior shoulder dislocation. There has only been one previous systematic review and meta-analysis by Dong et al. [20] in 2021, which looked at closed shoulder reduction techniques and concluded that scapular manipulation was the best method as it was the most successful, fastest, least painful, and had the shortest hospital stay. Our findings specifically show that self-reduction leads to a much higher success rate and less maximum pain on reduction. In either group, no acute problems were detected.

In terms of success rate, all studies were in favor of supervised self-reduction. In two studies [2, 14] self-reduction procedures have a substantially higher success rate compared to physician-assisted techniques, with an odds ratio of 2.71 (95% CI 1.25–5.58, P-value = 0.01). Reduction pain is seen as a key impediment to shoulder reduction due to its interaction with muscular relaxation, which increases the demand for analgesics and increases reduction difficulties [21]. Based on our meta-analysis, self-reduction methods lead to significantly reduced maximum pain with a mean difference of 1.98 (95% CI 1.24–2.72,) and P-value < 0.01. This is because the traction-induced pain exacerbates muscle contracture, making the reduction attempts difficult and poorly tolerated by patients [10, 22, 23]. This is critical in reducing analgesic use, decreasing reduction time, increasing patient satisfaction, and eventually increasing success rates.

According to two studies [8, 14], physician-assisted procedures need more time than self-reduction techniques, which may be attributed to increased pain in the physician-assisted group and the patient-centered approach of self-reduction techniques and individual participation in the process.

Self-reduction techniques can also be done without direct supervision. Chechik et al. [24] taught patients how to self-reduce anterior shoulder dislocation through a video link. In this study, BHM, Milch, and Stimson led to 53%, 55%, and 16% success rates, respectively, which were inferior to those with supervised reduction techniques [2, 8, 14, 19]. It appears that self-reduction approaches can be employed in the field of telemedicine.

The type of premedication utilized for reduction is especially important regarding success rate. Wirbel et al. [2] used 10 mg midazolam before reducing with the Kocher method and had a 64.2% success rate, whereas Turturro et al. [14] achieved a 98.3% success rate with just 1.6% of patients undergoing premedication with benzodiazepines and 45.9% not using any premedication. It could be explained by the patient’s central role in the self-reduction method and the benzodiazepine-induced dizziness and confusion.

Self-reduction procedures can be used even without initial radiographs to reduce treatment time and expenses when the doctor is confident in the diagnosis of anterior shoulder dislocation and none of the three criteria, are present [25–28].

This study has some limitations: Similar to other meta-analyses, our study has certain limitations regarding the search process. One such constraint is that the articles included in our analysis were limited to the period between 2014 and 2022, which may introduce a timeframe bias. Additional limitations encompass the restricted number of patients assessed, a high proportion of male patients, and the absence of assessment in elderly patients. In the included articles, younger patients enrolled in the self-reduction group, which may be a source of bias, even though a multivariate analysis was performed in the Marcano Fernandez et al. [8] study indicated that the confounding effect of age was negligible; finally, in two studies [8, 14] residents performed the reduction and the role of learning curves didn’t investigate; and third Wirbel et al. [2] used premedication.

We suggest future research use learning curves and randomized trial designs with larger sample sizes to understand each intervention’s merits and cons better.

In conclusion, none of these techniques led to complications in this systematic review, and we advocate self-reduction techniques for young patients with recurrent shoulder dislocation. It is essential to acknowledge that self-reduction techniques can be employed when radiographical techniques are unavailable, or clinicians are confident in their diagnosis. This can effectively minimize radiation exposure associated with radiography. Supervised self-reduction techniques have a greater success rate, lower pain, higher patient satisfaction, less reduction time, and reduced emergency department visits, and minimizing the use of analgesic drugs that can have adverse effects such as confusion and dizziness This effect enables these approaches applicable in healthcare settings outside of the emergency room and use as a telemedicine approach. Physician-assisted techniques are preferable when self-reduction techniques fail.

### Electronic supplementary material

Below is the link to the electronic supplementary material.

Supplementary Material 1

Supplementary Material 2

Supplementary Material 3

## Data Availability

All data generated or analyzed data in the study are included in this article.
