# Supplementary material for: Efficacy of supervised self-reduction vs. physician-assisted techniques for anterior shoulder dislocations: a systematic review and meta-analysis
Source: BMC Musculoskelet Disord. 2024 May 11;25:372. doi: 10.1186/s12891-024-07379-0 (PMC11088172; doi:10.1186/s12891-024-07379-0)
Supplement: Supplementary file 1 — Supplementary Material 1 [file 12891_2024_7379_MOESM1_ESM.docx]

## Characteristics of excluded studies

[Ordered by Date]

| Study ID | Reason for exclusion |
| --- | --- |
| Cofield 1985[1] | Enough data was not provided.  We tried to reach authors for data but were not successful. |
| Noordeen 1992[2] | Not a self-reduction technique |
| Johnson 1992[3] | Not a self-reduction technique |
| Boss 1993[4] | Enough data was not provided.  We tried to reach authors for data but were not successful. |
| Boss 1993[5] | Enough data was not provided.  We tried to reach authors for data but were not successful. |
| Ceroni 1997[6] | Enough data was not provided.  We tried to reach authors for data but were not successful. |
| Joy 2000[7] | Narrative review |
| Meyer-Rath 2001[8] | Enough data was not provided.  We tried to reach authors for data but were not successful. |
| Ito 2001[9] | Enough data was not provided.  We tried to reach authors for data but were not successful. |
| Baykal 2005[10] | Not a self-reduction technique |
| O’Connor 2006[11] | Not a self-reduction technique |
| Fernández-Valencia 2009[12] | Not a self-reduction technique |
| Hou 2009[13] | Enough data was not provided.  We tried to reach authors for data but were not successful. |
| Sayegh 2009[14] | Not a self-reduction technique |
| Ditty 2010[15] | Irrelevant participants |
| Dudkiewicz 2010[16] | Lack of control group |
| Pishbin 2011[17] | Not a self-reduction technique |
| Harnroongroj 2011[18] | Not a self-reduction technique |
| Maity 2012[19] | Not a self-reduction technique |
| Fang 2013[20] | Enough data was not provided.  We tried to reach authors for data but were not successful. |
| Gül 2014[21] | Not a self-reduction technique |
| Ghane 2014[22] | Not a self-reduction technique |
| Sapkota 2015[23] | Not a self-reduction technique |
| Bokor-Billmann 2015[24] | Not a self-reduction technique |
| Janitzky 2015[25] | Not a self-reduction technique |
| Marcano-Fernández 2015 | A protocol |
| Stafylakis 2016[26] | Lack of control group |
| Baden 2017[27] | Not a self-reduction technique |
| Su 2018[28] | Not a self-reduction technique |
| Mirafzal 2018 | A protocol |
| Dowson 2019[29] | Not a trial / Not a self-reduction technique |
| Chamseddine 2019[30] | Not a self-reduction technique |
| Lu 2019[31] | Enough data was not provided.  We tried to reach authors for data but were not successful. |
| Akcimen 2020[32] | Not a self-reduction technique |
| Chechik 2020[33] | Lack of control group |

# References

Uncategorized References

1. Cofield, R.H., B.F. Kavanagh, and F.J. Frassica, *Anterior shoulder instability.* Instr Course Lect, 1985. **34**: p. 210-27.

2. Noordeen, M.H., et al., *Anterior dislocation of the shoulder: a simple method of reduction.* Injury, 1992. **23**(7): p. 479-80.

3. Johnson, G., W. Hulse, and A. McGowan, *The Milch technique for reduction of anterior shoulder dislocations in an accident and emergency department.* Arch Emerg Med, 1992. **9**(1): p. 40-3.

4. Boss, A., P. Holzach, and P. Matter, *[A new self-repositioning technique for fresh, anterior-lower shoulder dislocation].* Helv Chir Acta, 1993. **60**(1-2): p. 263-5.

5. Boss, A., P. Holzach, and P. Matter, *[Analgesic-free self-reduction of acute shoulder dislocation].* Z Unfallchir Versicherungsmed, 1993. **Suppl 1**: p. 215-20.

6. Ceroni, D., H. Sadri, and A. Leuenberger, *Anteroinferior shoulder dislocation: an auto-reduction method without analgesia.* J Orthop Trauma, 1997. **11**(6): p. 399-404.

7. Joy, E.A., *Self-reduction of anterior shoulder dislocation.* Phys Sportsmed, 2000. **28**(11): p. 65-6.

8. Meyer-Rath, J.C., Gelbke, R., *Assisted self-reduction of anterior shoulder luxation.* Chirurgische Praxis, 2001. **58**(2): p. 271-279.

9. Ito, H., A. Takayama, and Y. Shirai, *Abduction-and-horizontal-adduction technique for reduction of acute anterior shoulder dislocations: a simple technique evaluated with radiographs.* Am J Orthop (Belle Mead NJ), 2001. **30**(3): p. 201-4.

10. Baykal, B., S. Sener, and H. Turkan, *Scapular manipulation technique for reduction of traumatic anterior shoulder dislocations: experiences of an academic emergency department.* Emerg Med J, 2005. **22**(5): p. 336-8.

11. O'Connor, D.R., et al., *Painless reduction of acute anterior shoulder dislocations without anesthesia.* Orthopedics, 2006. **29**(6): p. 528-32.

12. Fernández-Valencia, J.A., et al., *The Spaso technique: a prospective study of 34 dislocations.* Am J Emerg Med, 2009. **27**(4): p. 466-9.

13. Hou, Y.K., et al., *[Treatment of anterior shoulder dislocation with self-traction and self-reset].* Zhongguo Gu Shang, 2009. **22**(9): p. 711-2.

14. Sayegh, F.E., et al., *Reduction of acute anterior dislocations: a prospective randomized study comparing a new technique with the Hippocratic and Kocher methods.* J Bone Joint Surg Am, 2009. **91**(12): p. 2775-82.

15. Ditty, J., et al., *Safety and efficacy of attempts to reduce shoulder dislocations by non-medical personnel in the wilderness setting.* Wilderness Environ Med, 2010. **21**(4): p. 357-361.e2.

16. Dudkiewicz, I., et al., *Patients education of a self-reduction technique for anterior glenohumeral dislocation of shoulder.* J Trauma, 2010. **68**(3): p. 620-3.

17. Pishbin, E., E. Bolvardi, and K. Ahmadi, *Scapular manipulation for reduction of anterior shoulder dislocation without analgesia: results of a prospective study.* Emerg Med Australas, 2011. **23**(1): p. 54-8.

18. Harnroongroj, T., J. Wangphanich, and T. Harnroongroj, *Efficacy of gentle traction, abduction and external rotation maneuver under sedative-free for reduction of acute anterior shoulder dislocation: retrospective comparative study.* J Med Assoc Thai, 2011. **94**(12): p. 1482-6.

19. Maity, A., D.S. Roy, and B.C. Mondal, *A prospective randomised clinical trial comparing FARES method with the Eachempati external rotation method for reduction of acute anterior dislocation of shoulder.* Injury, 2012. **43**(7): p. 1066-70.

20. Fang, J., et al., *[Manipulation of superduct, adduction, rotation for the treatment of shoulder dislocation].* Zhongguo Gu Shang, 2013. **26**(1): p. 16-8.

21. Gül, M., et al., *Flexion-adduction-external rotation method for shoulder dislocations.* Acta Orthop Traumatol Turc, 2014. **48**(2): p. 164-8.

22. Ghane, M.R., et al., *Comparison between traction-countertraction and modified scapular manipulation for reduction of shoulder dislocation.* Chin J Traumatol, 2014. **17**(2): p. 93-8.

23. Sapkota, K., et al., *Comparison between external rotation method and milch method for reduction of acute anterior dislocation of shoulder.* J Clin Diagn Res, 2015. **9**(4): p. Rc01-3.

24. Bokor-Billmann, T.L., H; Kiffner, E; Goos, MF; Hopt, UT; Billmann, FG; Bokor-Billmann, Therezia; Lapshyn, Hryhoryi; Kiffner, Erhard; Goos, Matthias F.; Hopt, Ulrich T.; Billmann, Franck G., *Reduction of Acute Shoulder Dislocations in a Remote Environment: A Prospective Multicenter Observational Study.* WILDERNESS & ENVIRONMENTAL MEDICINE, 2015. **26**(3): p. 395-400.

25. Janitzky, A.A., et al., *Anterior Shoulder Dislocations in Busy Emergency Departments: The External Rotation Without Sedation and Analgesia (ERWOSA) Method May Be the First Choice for Reduction.* Medicine (Baltimore), 2015. **94**(47): p. e1852.

26. Stafylakis, D., S. Abrassart, and P. Hoffmeyer, *Reducing a Shoulder Dislocation Without Sweating. The Davos Technique and its Results. Evaluation of a Nontraumatic, Safe, and Simple Technique for Reducing Anterior Shoulder Dislocations.* J Emerg Med, 2016. **50**(4): p. 656-9.

27. Baden, D.N., et al., *Biomechanical reposition techniques in anterior shoulder dislocation: a randomised multicentre clinical trial- the BRASD-trial protocol.* BMJ Open, 2017. **7**(7): p. e013676.

28. Su, H., et al., *Hippocratic method for reduction of chronic locked anterior shoulder dislocations.* Orthopade, 2018. **47**(1): p. 67-72.

29. Dowson, P., *Shoulder Reduction Bench Project: improving care for patients with shoulder dislocations.* BMJ Open Qual, 2019. **8**(2): p. e000366.

30. Chamseddine, A.H., et al., *FARES method for reduction without medication of first episode of traumatic anterior shoulder dislocation.* Int Orthop, 2019. **43**(5): p. 1165-1170.

31. Lu, B.J., Z. Lu, and X.F. Shen, *[Treatment of anterior glenohumeral dislocations with manual reduction of WU medical school].* Zhongguo Gu Shang, 2019. **32**(12): p. 1165-1167.

32. Akcimen, M. and C. Bedel, *Comparison between new modified external rotation method and external rotation method for reduction of ASD.* Am J Emerg Med, 2020. **38**(5): p. 874-878.

33. Chechik, O., et al., *Self-reduction techniques taught via smartphone are effective for anterior shoulder dislocation: prospective randomized study.* Knee Surg Sports Traumatol Arthrosc, 2021. **29**(7): p. 2338-2341.
