# Supplementary material for: Efficacy of supervised self-reduction vs. physician-assisted techniques for anterior shoulder dislocations: a systematic review and meta-analysis
Source: BMC Musculoskelet Disord. 2024 May 11;25:372. doi: 10.1186/s12891-024-07379-0 (PMC11088172; doi:10.1186/s12891-024-07379-0)
Supplement: Supplementary file 2 — Supplementary Material 2 [file 12891_2024_7379_MOESM2_ESM.docx]

**Table S1.** Quality assessment of RCT studies.

| Author | 1 | 2 | 3 | 4 | 5 | 6 | 7 | 8 | 9 | 10 | 11 | 12 | 13 |
| --- | --- | --- | --- | --- | --- | --- | --- | --- | --- | --- | --- | --- | --- |
| Marcano-Fernandez 2018 | Y | Y | Y | N | N | Y | Y | NA | N | Y | N | Y | Y |
| Turturro 2014 | N | N | Y | N | N | UC | N | Y | NA | Y | Y | Y | Y |
| Chechik 2020 | Y | Y | Y | N | N | UC | Y | Y | NA | Y | N | Y | Y |
| Silva 2022 | N | N | Y | N | N | Y | Y | Y | NA | Y | N | Y | Y |

1. Was true randomization used for assignment of participants to treatment groups?

2. Was allocation to treatment groups concealed?

3. Were treatment groups similar at the baseline?

4. Were participants blind to treatment assignment?

5. Were those delivering treatment blind to treatment assignment?

6. Were outcomes assessors blind to treatment assignment?

7. Were treatment groups treated identically other than the intervention of interest?

8. Was follow up complete and if not, were differences between groups in terms of their follow up adequately described and analyzed?

9. Were participants analyzed in the groups to which they were randomized?

10. Were outcomes measured in the same way for treatment groups?

11. Were outcomes measured in a reliable way?

12. Was appropriate statistical analysis used?

13. Was the trial design appropriate, and any deviations from the standard RCT design (individual randomization, parallel groups) accounted for in the conduct and analysis of the trial?
