# Supplementary material for: Efficacy of supervised self-reduction vs. physician-assisted techniques for anterior shoulder dislocations: a systematic review and meta-analysis
Source: BMC Musculoskelet Disord. 2024 May 11;25:372. doi: 10.1186/s12891-024-07379-0 (PMC11088172; doi:10.1186/s12891-024-07379-0)
Supplement: Supplementary file 3 — Supplementary Material 3 [file 12891_2024_7379_MOESM3_ESM.docx]

Systematic Review Protocol

**Efficacy of self-reduction techniques for anterior shoulder dislocations**

# Topic as in PICOT format

**P**: Patients with anterior shoulder dislocation, either first or recurrent episodes, regardless of age, sex, and ethnicity

**I**: Self-reduction techniques, such as Boss-Holtzach-Matter, Milch, and Stimson

**C**: Other techniques rather than self-reduction techniques

**O**: Treatment Efficacy (Success rate, Pain during reduction, Reduction time, Recurrence rate, Patient subjective satisfaction)

Complications (Axillary artery or nerve injury, Fractures of the humerus, glenoid, or coracoid process, Rotator cuff injuries)

**T**: All types of papers excluding case reports, case series, letters, correspondents, and commentaries.

Search strategy

## Databases

## Medline/PubMed

( (“Shoulder Joint”[MeSH Terms] AND “Joint Dislocations”[MeSH Terms]) OR “Shoulder Dislocation"[MeSH Terms] OR “Shoulder dislocat*”[Title/Abstract] OR “Glenohumeral dislocat*”[Title/Abstract]) **AND** ( "Manipulation, Orthopedic"[Mesh] OR Self-reduc*[Title/Abstract] OR “Self reduc*”[Title/Abstract] OR Self-assisted[Title/Abstract] OR Self-relocation[Title/Abstract] OR Auto-reduction[Title/Abstract] OR Boss-Holtzach-Matter[Title/Abstract] OR Davos[Title/Abstract] OR Stimson[Title/Abstract] OR Milch[Title/Abstract] ) **AND** ( “Treatment Outcome”[MeSH Terms] OR efficacy[Title/Abstract] OR effective*[Title/Abstract] OR Recurren*[Title/Abstract] OR Satisf*[Title/Abstract] OR Pain[Title/Abstract] OR Success [Title/Abstract] )

## Scopus

( TITLE-ABS-KEY ((“Shoulder Joint” AND “Joint Dislocat*”) OR "Shoulder dislocat*" OR "Glenohumeral dislocat*" ) ) **AND** ( TITLE-ABS-KEY ( "Self-reduc*" OR "self-assisted" OR "self-relocation" OR "auto-reduction" OR "Boss-Holtzach-Matter" OR "davos" OR "Stimson" OR "Milch" ) ) **AND** ( TITLE-ABS-KEY ( “outcom*” OR "efficacy" OR "effective*" OR "Recurren*" OR "satisf*" OR "Pain" OR “success”) )

## CENTRAL

#1 MESH DESCRIPTOR Shoulder Joint EXPLODE ALL TREES

#2 MESH DESCRIPTOR Joint Dislocations EXPLODE ALL TREES

#3 #1 AND #2

#4 MESH DESCRIPTOR Shoulder Dislocation EXPLODE ALL TREES

#5 ("Shoulder dislocation" ):TI,AB,KY

#6 ("Glenohumeral dislocation"):TI,AB,KY

#7 #3 OR #4 OR #5 OR #6

#8 ("Self-reduction" OR "self-assisted" OR "self-relocation" OR "auto-reduction" OR "Boss-Holtzach-Matter" OR "davos" OR "Stimson" OR "Milch"):TI,AB,KY

#9 MESH DESCRIPTOR Manipulation, Orthopedic EXPLODE ALL TREES

#10 #8 OR #9

#11 MESH DESCRIPTOR Treatment Outcome EXPLODE ALL TREES

#12 ("efficacy" OR "effectiveness" OR "Recurrence" OR "satisf" OR "Pain" OR ?success?):TI,AB,KY

#13 #11 OR #12

## #14 #7 AND #10 AND #13Web of Science

( (TS=(“Shoulder Joint”) AND TS=(“Joint Dislocat*”)) OR TS=(“Shoulder Dislocat*”) OR TS=(“Glenohumeral Dislocat*”) ) **AND** ( TS=(“self-reduc*”) OR TS=(“Self-assisted”) OR TS=(“Self-relocation”) OR TS=(“Auto-reduction”) OR TS=(Stimson) OR TS=(Milch) OR TS=(Boss-Holtzach-Matter) OR TS=(Davos) ) **AND** ( TS=(Outcome$) OR TS=(Efficacy) OR TS=(Effective*) OR TS=(Recurren*) OR TS=(satisf*) OR TS=(Pain) OR TS=(success) )

## Restrictions

No language, filter or date restriction

|  | **Shoulder Dislocation** | **Self-Reduction** | **Treatment outcome** |
| --- | --- | --- | --- |
| Mesh | Shoulder Dislocation  Shoulder Joint  Joint Dislocations | Manipulation, Orthopedic  /therapy | Treatment Outcome |
|  | Shoulder dislocat* | Self-reduc* | efficacy |
|  | Glenohumeral dislocat* | Self-assisted | Effective* |
|  |  | Self-relocation | Success |
|  |  | Auto-reduction | Recurren* |
|  |  | Boss-Holtzach-Matter | satisf* |
|  |  | Davos | Pain |
|  |  | Stimson |  |
|  |  | Milch |  |
|  |  |  |  |
